# The association of demodex infestation with pediatric chalazia

**DOI:** 10.1186/s12886-022-02261-w

**Published:** 2022-03-16

**Authors:** Jing Huang, Meng-Xiang Guo, Dao-Man Xiang, Li-Feng Yan, Ying Yu, Ling Han, Jian-Xun Wang, Xiao-He Lu

**Affiliations:** 1 Department of Ophthalmology, Zhujiang Hospital, Southern Medical University, Guangzhou, 510282 Guangdong Province China; 2 Department of Ophthalmology, Guangzhou Women and Children’s Medical Center, Guangzhou, 510623 Guangdong Province China

**Keywords:** Chalazia, Demodex, Demodicosis, Pediatric, Meibomian gland cyst

## Abstract

**Purpose:**

This study aimed to investigate the association of *Demodex* infestation with pediatric chalazia.

**Methods:**

In a prospective study, 446 children with chalazia and 50 children with non-inflammatory eye disease (controls) who underwent surgical treatment were enrolled from December 2018 to December 2019. Patient ages ranged from 7 months to 13 years old. All patients underwent eyelash sampling for light microscope examination, and statistical correlation analysis between *Demodex* infestation and chalazia, including the occurrence, recurrence, and course of disease, morphological characteristics, and meibomian gland dysfunction (MGD) in chalazia patients was performed.

**Results:**

*Demodex* was found in 236 (52.91%) patients with chalazia and zero control patients. Demodicosis was significantly more prevalent in chalazia patients than the control group (*P* < 1 × 10^− 14^). Recurrent chalazia (*P* = 0.006) and skin surface involvement (*P* = 0.029) were highly correlated with *Demodex* infestation. Demodicosis was also associated with multiple chalazia (*P* = .023) and MGD(*P* = .024). However, *Demodex* infestation was comparable in the course of disease (*P* = 0.15), seasonal change (*P* = 0.68) and blepharitis subgroups (*P* = 0.15). Within the group of chalazia patients who underwent surgical removal of cysts, 4 (0.9%) patients with concurrent demodicosis experienced recurrence.

**Conclusions:**

*Demodex* infestation was more prevalent in pediatric chalazia patients than healthy children, and was associated with recurrent and multiple chalazia. Demodicosis should be considered as a risk factor of chalazia. In children with chalazia, *Demodex* examination and comprehensive treatment of *Demodex* mites should be applied to potentially prevent recurrence.

## Introduction

Chalazia is a common lid disease that is characterized by chronic granulomatous inflammation of meibomian glands, and is prone to recurrence. Occasionally, cysts on the skin surface may spontaneously rupture, leaving obvious scarring. Preventing recurrence and reducing treatment costs remains challenging. Previous studies have found that chronic conjunctivitis, blepharitis, excessive secretion from sebaceous or sweat glands, and vitamin A deficiency are common causes of chalazia, but controlling these conditions did not reduce the incidence or recurrence of chalazia [1, 2]. In recent years, the pathogenicity of ocular demodicosis was emphasized, with studies indicating that Demodex significantly impacted the onset of anterior blepharitis, including refractory and scaly blepharitis [3, 4]. Posterior blepharitis is associated with the meibomian glands, but has not been the focus of research. We aimed to analyze the association of *Demodex* infestation with chalazia in pediatric patients.

## Materials and Methods

### Patients

This study was approved by the ethics committee of Guangzhou Women and Children’s Medical Center and all methods in this research were performed in accordance with the relevant guidelines and regulations. In this Prospective, observational, comparative designed study, all patients were randomly selected, and informed consent was obtained from their parents/guardians prior to enrollment. The study consisted of 496 pediatric patients with ocular diseases who underwent surgery between December 2018 and December 2019. The average age was 3.36 ± 1.61 years (range, 7 months to 13 years). The patients were divided into a study group of 446 chalazia patients, and an age- and gender-matched control group of 50 patients who underwent surgery for strabismus or orbital dermoid cysts (38 and 12 cases, respectively). Inclusion criteria were age under 14 years, and no acute inflammation, to avoid potential microbial infections. Patients taking immunosuppressants, and those with rosacea based on medical history and clinical signs, were excluded because of the potential association with *Demodex* infection [5–7].

### Eyelash sampling and microscopic *Demodex* examination

**Fig. 1:**
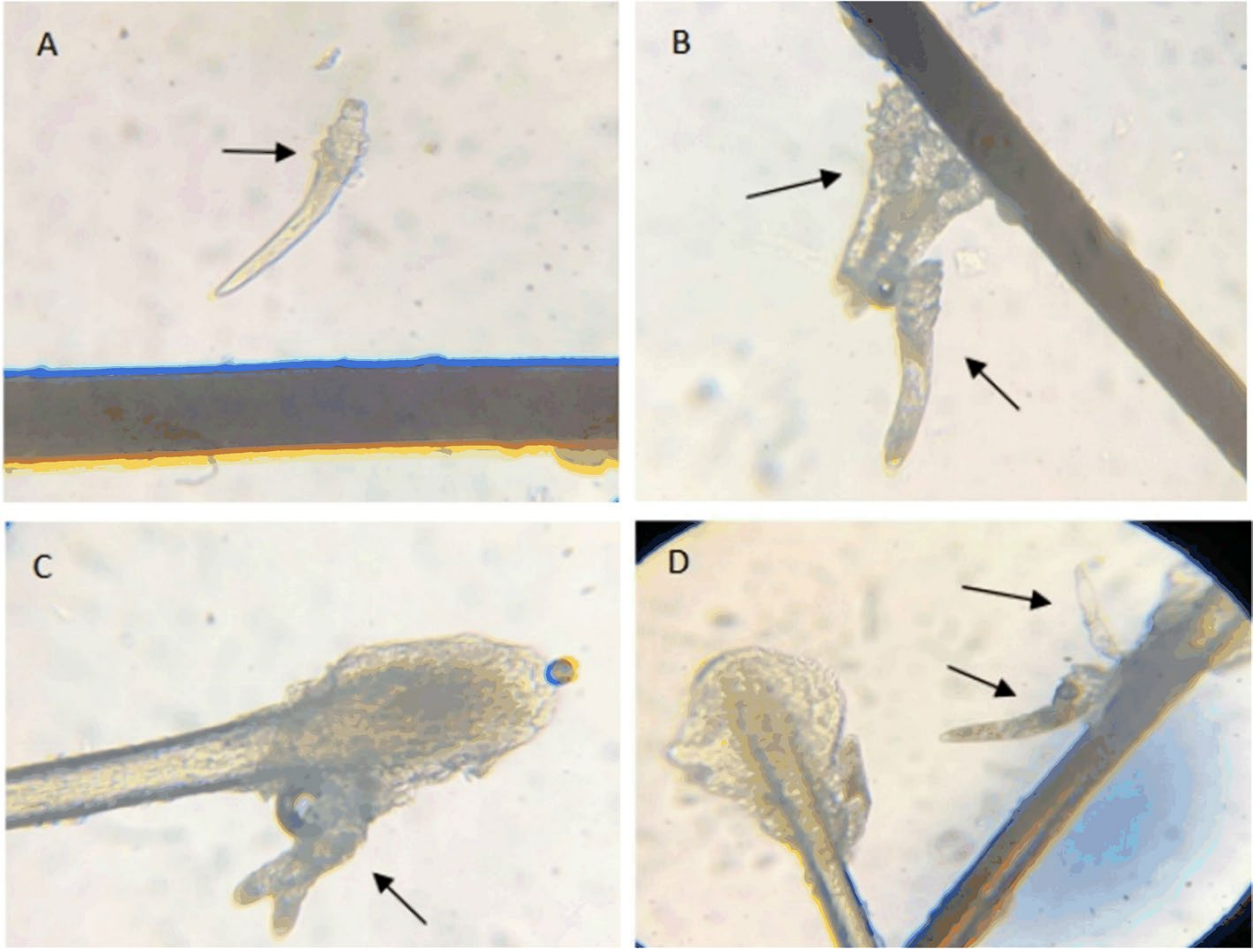
Morphology of *Demodex* at the root of eyelashes under optical microscope (100X). **A** *Demodex* folliculorum nymph, with a thin body, has differentiated into 4 pairs of feet. **B** *Demodex* folliculorum adults. **C** *Demodex* brevis. **D** *Demodex* folliculorum adults and larvae

Eyelash sampling and microscopic *Demodex* examination were performed as previously described [8–11]. In brief, 3 lashes were epilated from each eyelid under general anesthesia before surgery. Eyelashes located near meibomian gland cysts or where cylindrical dandruff was visible, and where meibomian glands were dilated were prioritized to increase the chance of detection. The eyelashes were placed separately on a glass slide and mounted with a coverslip; 1 drop of saline solution was applied to the edge of the coverslip before microscopic examination. All samples were taken by the same operator and *Demodex* detection was performed by an independent masked technician who had no knowledge about each patient’s clinical information. At least one *Demodex* mite (including adult, larva, nymph or egg) in a 100X magnified field was considered a positive result (Fig. 1).

### Treatment of *Demodex* infestation and follow-up

Considering the biological pathogenicity and the contagiousness of *Demodex*, the children with demodicosis were recommended for treatment in both eyes. The comprehensive treatment for demodicosis was initiated 10 days after chalazia removal surgery. A warm compress (warm towel or hot compress eyeshade, 40–45 °C, 10–15 min) was applied first, followed by using a terpinen-4-ol wipe to scrub the lash roots from one end to the other in one stroke, which was repeated for at least 30 s. Metronidazole gel was manually applied on the lash roots with the eyes closed. This procedure was repeated twice daily. Routine assessment was performed monthly, with initiation of a second course of treatment if *Demodex* mites persisted.

### Statistical analyses

All statistical analyses were performed using SPSS software, version 19.0 (SPSS, Inc. Chicago, Illinois, USA). Continuous data are reported as the mean ± standard deviation and discrete data are expressed as rates (%). Multivariate logistic regression modeling was used to analyze the correlation strength between relevant factors and *Demodex* infestation. The Pearson chi-square test was used for comparative and correlative analyses between subgroups. The Fisher’s exact test was used for data with low prediction frequency to avoid statistical bias. *P* < 0.05 was considered statistically significant.

## Results

1. *Demodex* infestation was present in significantly more chalazia patients (52.19%) than control patients (0%) (*P* < 1 × 10^− 14^).

2. Chalazia group:

- 2.1 Relevant factors were divided into subgroups (Table 1), and *Demodex* infestation was comparable when analyzed in terms of gender(*P* = 0.84) and age(*P* = 0.17). *Demodex* was also associated with recurrent (*P* = 0.002), multiple (*P* = 0.023), and skin nodule (*P* = 0.001) chalazia, and MGD (*P* = 0.024). The MGD subgroup specifically referred to meibomian glands that appeared healthy, with the exception of cyst formation, such as meibomian gland expansion, viscosity of the meibomian gland secretion increased, secretion changes from transparent to yellowish-white and ooze toothpaste-like excretion when squeezing the meibomian gland. However, *Demodex* infestation was comparable in unilateral or bilateral (*P* = 0.59), course of disease (*P* = 0.15), seasonal changes (*P* = 0.68), and anterior blepharitis (*P* = 0.15).
- 2.2 Results of the multivariate logistic regression analysis are shown in Table 2. Recurrent chalazia or skin surface involvement were highly correlated with *Demodex* infestation (*P* = 0.006 and *P* = 0.029, respectively).
- 2.3 Patients with demodicosis who underwent 1 month of *Demodex* treatment and then were lost to follow-up (*n* = 69) were excluded, and the remaining 167 patients were followed up for successful *Demodex* treatment. The average treatment duration was 1.74 ± 0.78 months (range from 1 to 5 months; 74 patients for 1 month, 64 for 2 months, 28 for 3 months, 1 for 5 months). Four recurrences (0.9%) were observed in chalazia patients after surgical removal. Recurrence occurred in patients with common characteristics such as young age (29–48 months), *Demodex* infection, MGD, and multiple cysts. In this study, only 23 chalazia patients (9.75%) had mites in ≥2/12 lashes.

**Table 1:** Comparison between the subgroups

Subgroups	Total cases	*Demodex* infestation cases (%)
Gender		
Male	225	118(52.44%)
Female	221	118(53.39%)
Age(year)		
≤6	425	226(53.18%)
>6	21	10(47.62%)
Course of disease(week)		
≤2	19	7(36.84%)
>2	427	229(53.63%)
Affected eye		
Unilateral	111	56(50.45%)
Bilateral	335	179(53.43%)
Recurrent		
Negative	422	216(51.18%)
Positive	24	20(83.33%)
Cyst count		
Single	39	11(28.21%)
Multiple	407	225(55.28%)
Skin nodule		
Positive	305	177(58.03%)
Negative	141	59(41.84%)
MGD		
Positive	158	95(60.13%)
Negative	288	141(48.96%)
Anterior Blepharitis		
Positive	12	9(75%)
Negative	434	227(52.3%)
Season		
Winter half year	293	153(52.22%)
Summer half year	153	83(54.25%)

^Guangdong province belongs to the East Asian monsoon region with central subtropical, south subtropical and tropical climates from north to south. Winter half year is October to April and summer half year is May to September^

**Table 2:** Multivariate logistic regression analysis of Demodex infestation with relevant factors

Variable	Variable assignments	B	***P***	OR(95%CI)
Gender	female =0	-0.093	0.64	0.911[0.617,1.347]
	male =1			
Age	≤6=0	-0.406	0.29	0.666[0.315,1.408]
	>6=1			
Course of disease	≤2=0	0.858	0.11	2.358[0.837,6.646]
	>2=1			
Nodule location	conjunctival surface=0	0.498	0.029^*^	1.645[1.054,2.568]
	skin surface=1			
Cyst count	single=0	0.766	0.06	2.152[0.980,4.724]
	multiple=1			
MGD	negative=0	-0.315	0.14	0.729[0.479,1.111]
	positive=1			
Anterior Blepharitis	negative=0	-0.938	0.18	0.391[0.100,1.532]
	positive=1			
Recurrent	negative=0	1.598	0.006^**^	4.942[1.589,15.369]
	positive=1			

^B is the regression coefficient, OR is the ratio of dominance, CI is the confidence interval. Multivariate Logistic regression, [Recurrence]** *P*<0.01; [Skin nodule]* *P*<0.05^

## Discussion

*Demodex* mites are common in nature, and people are generally susceptible to them. There are two species of *Demodex* parasites that can infest human skin. *Demodex follicularis* lives in eyelash follicles, whereas *Demodex brevis* burrows into meibomian and sebaceous glands of the eyelid [12]. Both species *Demodex* were clearly observed in patients with demodicosis and chalazia. Most patients with demodicosis are asymptomatic mite carriers. Recently, it has been reported that demodicosis was prevalent in cases of blepharitis, and that *Demodex* mites played an important role in chronic inflammation of the skin, hair follicles, and glands of the eyelid [3]. Although the mechanism by which *Demodex* induce pathogenic damage is unclear, it likely involves direct damage from *Demodex* mites and their metabolites, delayed hypersensitivity induced by these metabolites, local infiltration of inflammatory cells, and secondary infection of pathogenic microorganisms, especially bacteria [8, 13].

Chalazia is common in pediatric patients, and presents with multiple and recurrent chalazia. Chalazia exhibits poor treatment coordination and is difficult to prevent. This study showed that *Demodex* infestation was more prevalent in patients with chalazia than the control group. Furthermore, demodicosis was not found in the control group. Demodicosis is commonly age dependent [11, 14–16], with more frequent detection in individuals over 71 years old than in children between 3 and 15 years old [11]. This was consistent with the observations that demodicosis was rare in healthy children [11, 17, 18], and suggesting that *Demodex* infestation of pediatric patients is a risk factor for chalazia.

Previous studies [2, 19] commonly used the presence of mites to determine *Demodex* infection. Even though *Demodex* mites were counted in some articles, the mite count was not used as the diagnostic criterion. There have not, to our knowledge, been specific studies in children with demodicosis, and the diagnosis and treatment for demodicosis in domestic populations are as follow: 2 mites/3 lashes in each eyelid is suspiciously positive and ≥ 3 mites/3 lashes is definitively positive, requiring clinical treatment [11]. In addition, *Demodex* lives in eyelash follicles, meibomian glands, and sebaceous glands [12]. Any mites attached to the eyelash root are removed simultaneously when eyelashes are removed for sampling, but it is important to consider that *Demodex* mites also lay eggs in the eyelash follicle, indicating that there may be more *Demodex* present than what is observed in the eyelash sample. Because demodicosis is not as common in the pediatric population, and mite count was not consistently correlated with the severity of chalazia [2, 6, 20, 21], presence of *Demodex* mites should be taken seriously. In our study, demodicosis with 2–3 mites/12 lashes contrived only 9.75% of chalazia patients with *Demodex* infestation. In pediatric chalazia, microscopic examination for *Demodex* mites should especially careful to avoid missing a diagnosis. In addition, if there is ≥1 mite/12 lashes, it is recommended that the patient be treated as *Demodex* positive and undergo intervention.

In this study, it was found that *Demodex* mites were more frequent in patients with recurrent chalazia and those with skin surface involvement. Moreover, ocular demodicosis was significantly more prevalent in patients with recurrent, multiple, and MGD chalazia, which matched the pathogenic role of mites in meibomian and sebaceous glands [13, 17, 19, 22]. Our results showed that demodicosis was more prevalent in chalazia patients regardless of blepharitis, similar to previously studies suggesting that demodicosis was associated with blepharitis [3, 4, 18] and chalazia [2, 13, 18, 19, 22]. These results also suggest that *Demodex* infestation could be an independent risk factor of chalazia after adjusting for the effects of blepharitis [23].

*Demodex* mites often utilize host cells and their metabolites, sebaceous gland secretions, sebum, and keratin as sources of nutrients. Changes in the local microenvironment of the eyelid caused by chalazia are conducive to *Demodex* parasitism, and demodicosis could worsen the manifestation of chalazia and cause its recurrence [8, 13, 15, 23]. This highlights the importance of mite treatment.

Recurrent chalazia is reported in 17–25% of affected children, which is more common than in the adult population [8, 24]. We observed that only 0.9% of pediatric patients with chalazia required re-operation for recurrence after comprehensive treatment for *Demodex* infestation. These results suggest that eradication of *Demodex* may be an effective method for preventing recurrence. Both 2% metronidazole ointment and Tea tree oil are reported as effective alternatives for treatment of *Demodex* infestation [25]. Terpinen-4-ol, which is the most active ingredient of tea tree oil, is currently the treatment of choice for pediatric demodicosis because it has fewer side effects [26, 27].

*Demodex* mites typically complete one generation of their life cycle in 14–15 days [8]. We recommend comprehensive treatment [28] for mites because children are often reluctant to cooperate with treatment. The course of treatment is generally 1 to 3 months, encompassing several *Demodex* life cycles [29].

In addition, ocular discomfort was often difficult to interpret due to difficulty for children to describe the sensation, causing complaints to be overlooked [30]. In order to prevent development of demodicosis, it is important for children and their parents to maintain good ocular hygiene, including applying warm compresses and contact-isolation of demodicosis to control ocular *Demodex* infection [23, 31].

This study had several associated limitations. Although adult or larvae *Demodex* were observed, *Demodex folliculorum* and *Demodex brevis* were not recorded separately for further study of which species was more prevalent in pediatric demodicosis with chalazia. Sampling the eyelash in the correct places is essential for microscopic *Demodex* detection, and there may be a non-invasive alternative, such as in vivo confocal microscopy, to sample eyelashes for *Demodex* detection, which could potentially avoid the failure to remove *Demodex* completely during epilation. This would also be more conducive to the review of pediatric patients. Since metronidazole gel has been reported to be both effective and safe in the treatment of demodicosis, comparing the effectiveness of different anti-mite treatments may be beneficial.

The majority of chalazia patients, especially those with recurrent and multiple chalazia, suffered more from demodicosis than healthy children. Demodicosis should be considered as a risk factor of chalazia. In children with chalazia, *Demodex* examination and comprehensive treatment of *Demodex* mites should be applied to potentially prevent recurrence.

## Data Availability

The datasets used and analysed during the current study are available from the corresponding author on reasonable request.
